# Effects of dapagliflozin and dapagliflozin-saxagliptin on erythropoiesis, iron and inflammation markers in patients with type 2 diabetes and chronic kidney disease: data from the DELIGHT trial

**DOI:** 10.1186/s12933-023-02027-8

**Published:** 2023-11-28

**Authors:** Akihiko Koshino, Brendon L. Neuen, Niels Jongs, Carol Pollock, Peter J. Greasley, Eva-Marie Andersson, Ann Hammarstedt, Cecilia Karlsson, Anna Maria Langkilde, Takashi Wada, Hiddo J.L. Heerspink

**Affiliations:** 1 Department of Clinical Pharmacy and Pharmacology, University of Groningen, Groningen, the Netherlands; 2 Department of Nephrology and Laboratory Medicine, Kanazawa University, Ishikawa, Japan; 3 The George Institute for Global Health, UNSW Sydney, Sydney, Australia; 4 Kolling Institute of Medical Research, Sydney Medical School, University of Sydney, Sydney, Australia; 5 Royal North Shore Hospital, St Leonards, NSW Australia; 6 BioPharmaceuticals Research and Development, AstraZeneca, Gothenburg, Sweden

**Keywords:** Anemia, Ferritin, Hematopoiesis, Interleukin-6, Iron metabolism, Monocyte chemoattractant protein-1, Sodium glucose co-transporter 2 inhibitors, Transferrin

## Abstract

**Background:**

This post-hoc analysis of the DELIGHT trial assessed effects of the SGLT2 inhibitor dapagliflozin on iron metabolism and markers of inflammation.

**Methods:**

Patients with type 2 diabetes and albuminuria were randomized to dapagliflozin, dapagliflozin and saxagliptin, or placebo. We measured hemoglobin, iron markers (serum iron, transferrin saturation, and ferritin), plasma erythropoietin, and inflammatory markers (urinary MCP-1 and urinary/serum IL-6) at baseline and week 24.

**Results:**

360/461 (78.1%) participants had available biosamples. Dapagliflozin and dapagliflozin-saxagliptin, compared to placebo, increased hemoglobin by 5.7 g/L (95%CI 4.0, 7.3; p < 0.001) and 4.4 g/L (2.7, 6.0; p < 0.001) and reduced ferritin by 18.6% (8.7, 27.5; p < 0.001) and 18.4% (8.7, 27.1; p < 0.001), respectively. Dapagliflozin reduced urinary MCP-1/Cr by 29.0% (14.6, 41.0; p < 0.001) and urinary IL-6/Cr by 26.6% (9.1, 40.7; p = 0.005) with no changes in other markers.

**Conclusions:**

Dapagliflozin increased hemoglobin and reduced ferritin and urinary markers of inflammation, suggesting potentially important effects on iron metabolism and inflammation.

**Trial registration:**

ClinicalTrials.gov NCT02547935.

**Supplementary Information:**

The online version contains supplementary material available at 10.1186/s12933-023-02027-8.

## Background

Anemia is common in patients with chronic kidney disease (CKD), particularly those with type 2 diabetes [1, 2]. Decreased erythropoietin (EPO) synthesis and abnormal iron metabolism caused by inflammation are important contributors to anemia in patients with CKD [1].

Sodium glucose co-transporter 2 (SGLT2) inhibitors reduce CKD progression and the risk of kidney failure in patients with type 2 diabetes and CKD [3]. Although the underlying mechanism of kidney protection with SGLT2 inhibitors is multifactorial and incompletely understood, effects on hematopoiesis and inflammation are potentially related to their benefit on clinical outcomes [4–6]. In patients with heart failure or type 2 diabetes, SGLT2 inhibitors reduced transferrin saturation (TSAT), ferritin, and hepcidin and transiently increased EPO, suggesting SGLT2 inhibition may facilitate iron utilization and promote erythropoiesis [7–9]. Clinical trials with SGLT2 inhibitors also reported reductions in circulating and urinary inflammatory mediators, including monocyte chemoattractant protein-1 (MCP-1) and Interleukin-6 (IL-6) [10–12]. However, effects of SGLT2 inhibitors on iron metabolism and inflammation and how these are associated in patients with type 2 diabetes and CKD is lacking.

In this post-hoc analysis of the DELIGHT trial [13], we therefore evaluated the effects of the SGLT2 inhibitor dapagliflozin, with and without the dipeptidyl peptidase-4 (DPP-4) inhibitor saxagliptin compared to placebo, on markers of erythropoiesis, iron metabolism, and inflammation in patients with type 2 diabetes and CKD. We also assessed correlations between markers of iron metabolism and inflammation.

## Methods

### Patients and study design

DELIGHT was a double-blind, placebo-controlled, multicenter trial that enrolled 461 patients from nine countries. The study was conducted from July 2015 to May 2018. Methods and results were previously published [13]. In short, DELIGHT trial assessed albuminuria lowering effect of dapagliflozin with and without saxagliptin. After a 4-week run-in period, participants were randomly assigned to 24 weeks treatment with 10 mg dapagliflozin, a combination of 10 mg dapagliflozin and 2.5 mg saxagliptin or matching placebo according to pre-enrolment glucose-lowering therapy strata. Eligible participants were aged ≥ 18 years and diagnosed with type 2 diabetes. Participants were required to have urinary albumin-creatinine ratio (UACR) of 30-3500 mg/g, eGFR of 20–80 ml/min/1.73 m², and HbA1c of 7.0–11.0% at screening.

Excluding criteria included a diagnosis of type 1 diabetes and non-diabetic kidney disease. Patients with hemoglobin < 9 g/dL before screening or long-term use of glucocorticoids were also excluded. All participants provided written informed consent. Participants were offered separate and optional informed consent to collect additional blood or urine samples for future biomarker research. DELIGHT trial was conducted according to the Declaration of Helsinki, registered with clinicaltrials.gov (NCT02547935), and approved by an ethics committee at each site.

### Biomarker assessment

Urine and blood samples for exploratory biomarker research were obtained at baseline and week 24. Samples were stored at -80 °C until measurement. We measured the following iron and erythropoiesis markers: serum iron, ferritin, transferrin, and plasma EPO. TSAT was calculated with iron and transferrin concentrations [14]. We measured the following markers of kidney and systemic inflammation: urinary MCP-1, urinary and serum IL-6. Urinary biomarkers were indexed to urinary creatinine (Cr) concentration to adjust for hydration status. Hemoglobin and hematocrit levels were measured at baseline, weeks 1 and 4, and every 4 weeks thereafter according to the study protocol.

### Statistical analysis

Baseline characteristics were reported in n (%) for categorical variables and in mean (SD) or median (IQR) for continuous variables. Biomarkers were log-transformed in the following analyses. We calculated mean percentage changes (95% CI) in biomarkers from baseline to week 24 by treatment allocation. Estimations were performed with analysis of covariance adjusted for randomization strata (insulin, metformin, sulfonylurea derivatives, thiazolidinediones, or other treatment-based regimens) and baseline value. Absolute mean changes (95% CI) in hemoglobin and hematocrit over time were assessed with repeated-measures models using a restricted maximum likelihood. The model consists of randomization strata, treatment allocation, visit and treatment-by-visit interaction as fixed effects, and baseline value as a covariate and baseline-by-visit interaction.

We calculated Pearson’s correlation coefficients to determine the relationship between markers of inflammation, iron metabolism and erythropoiesis at baseline. We also assessed correlations between changes in these markers by randomized treatment.

We considered p-values ≤ 0.05 as statistically significant. All analyses were performed with R Statistical Software version 4.2.1 (R Foundation for Statistical Computing, Vienna, Austria).

## Results

Of 461 participants, 360 (78.1%) had available biosamples for the current study. Baseline characteristics of subjects were similar to the overall DELIGHT population and were balanced between treatment allocation (Table 1). Correlation coefficients between inflammation markers and iron parameters and erythropoietin at baseline are shown in Additional file 1: Table S1. Serum IL-6 was negatively correlated with iron concentration (r= -0.18, p < 0.001) and TSAT (r= -0.14, p = 0.008). Urinary IL-6/Cr and erythropoietin did not correlate with any markers assessed.

**Table 1:** Baseline characteristics of participants with available biosamples and those in the entire trial

	Participants with available biosamples			
	Placebo	Dapagliflozin	Dapagliflozin-saxagliptin	Entire DELIGHT trial
	N = 119	N = 115	N = 126	N = 448
Age – year	64.1 (8.7)	65.2 (8.2)	63.4 (9.1)	64.4 (8.8)
Women– no. (%)	32 (26.9)	35 (30.4)	35 (27.8)	131 (29.2)
Race – no. (%)				
White	55 (46.2)	47 (40.9)	69 (54.8)	196 (43.8)
Black	8 (6.7)	6 (5.2)	5 (4.0)	26 (5.8)
Asian	37 (31.1)	49 (42.6)	40 (31.7)	177 (39.5)
Other	19 (16.0)	13 (11.3)	12 (9.5)	49 (10.9)
Diabetes duration - years	17.2 (9.4)	17.1 (7.9)	17.6 (7.9)	17.9 (8.5)
Body mass index – kg/m^2^	30.7 (5.5)	30.2 (5.4)	30.9 (5.4)	30.5 (5.4)
Systolic BP – mm Hg	140.6 (19.3)	138.7 (15.9)	139.5 (17.7)	139.2 (17.8)
Diastolic BP – mm Hg	76.0 (11.4)	77.1 (9.7)	77.4 (10.5)	76.6 (10.6)
Hematocrit – %	39.5 (5.4)	40.7 (4.8)	40.6 (5.4)	40.1 (5.1)
Hemoglobin – g/L	129.8 (18.1)	133.7 (16.4)	133.2 (18.1)	132.0 (17.3)
HbA1c - %	8.6 (1.2)	8.3 (1.0)	8.2 (1.0)	8.4 (1.1)
LDL cholesterol – mmol/L	2.3 (0.9)	2.3 (1.0)	2.2 (0.9)	2.3 (0.9)
HDL cholesterol – mmol/L	1.2 (0.4)	1.2 (0.3)	1.2 (0.4)	1.2 (0.4)
eGFR – mL/min/1.73 m^2^	46.8 (13.2)	48.8 (12.4)	48.4 (12.6)	49.0 (13.2)
UACR – mg/g (IQR)	283.7 (99.0, 986.8)	262.1 (64.5, 746.5)	222.2 (70.0, 786.8)	246.0 (73.0, 887.4)
Concomitant medication – no. (%)				
ESAs	1 (0.8)	1 (0.9)	0 (0.0)	2 (0.4)
Iron preparation	13 (10.9)	6 (5.2)	7 (5.6)	40 (8.9)
Iron and inflammation markers (IQR)				
Iron – µmol/L ^†^	13.2 (11.1, 17.5)	13.9 (10.4, 16.8)	13.3 (11.1, 16.8)	
Transferrin – mg/dL ^†^	2.5 (2.3, 2.8)	2.5 (2.3, 2.8)	2.4 (2.3, 2.8)	
TSAT – % ^†^	21.3 (16.9, 27.3)	21.6 (16.2, 27.7)	21.9 (17.9, 26.5)	
Ferritin – µg/L ^†^	125.0 (65.5, 256.0)	112.5 (67.8, 230.8)	116.0 (62.5, 196.0)	
Erythropoietin – pg/mL ^‡^	79.8 (59.6, 118.2)	82.9 (59.8, 123.2)	79.3 (61.9, 104.3)	
Urinary MCP-1/Cr – ng/g ^§^	169.8 (100.4, 278.4)	134.5 (94.3, 287.4)	142.0 (80.7, 245.2)	
Urinary IL-6/Cr – ng/g ^§^	0.7 (0.3, 1.5)	0.6 (0.4, 1.6)	0.6 (0.3, 1.4)	
Serum IL-6 – pg/mL ^†^	1.1 (0.6, 1.8)	1.0 (0.7, 1.6)	1.0 (0.6, 1.7)	

Patients with any biomarker measurement at both baseline and week 24 were included

^†^ 20 patients (8 in the placebo group; 5 in the dapagliflozin group; 7 in the dapagliflozin-saxagliptin group) did not have serum biomarker (Iron, Transferrin, TSAT, Ferritin and IL-6) measurements

^‡^ 16 patients (5 in the placebo group; 5 in the dapagliflozin group; 6 in the dapagliflozin-saxagliptin group) did not have available erythropoietin concentration

^§ 7^1 patients (28 in the placebo group; 21 in the dapagliflozin group; 22 in the dapagliflozin-saxagliptin group) did not have urinary biomarker (MCP-1/Cr and IL-6/Cr) measurements

BP, blood pressure; Cr, creatinine; eGFR, estimated glomerular filtration ratio; ESAs, Erythropoiesis stimulating agents; HbA1c, glycated hemoglobin; HDL, high-density lipoprotein; LDL, Low-density lipoprotein; MCP-1, monocyte chemoattractant protein; IL-6, interleukin-6; TSAT, transferrin saturation; UACR, urinary albumin-creatinine ratio

**Fig. 1:**
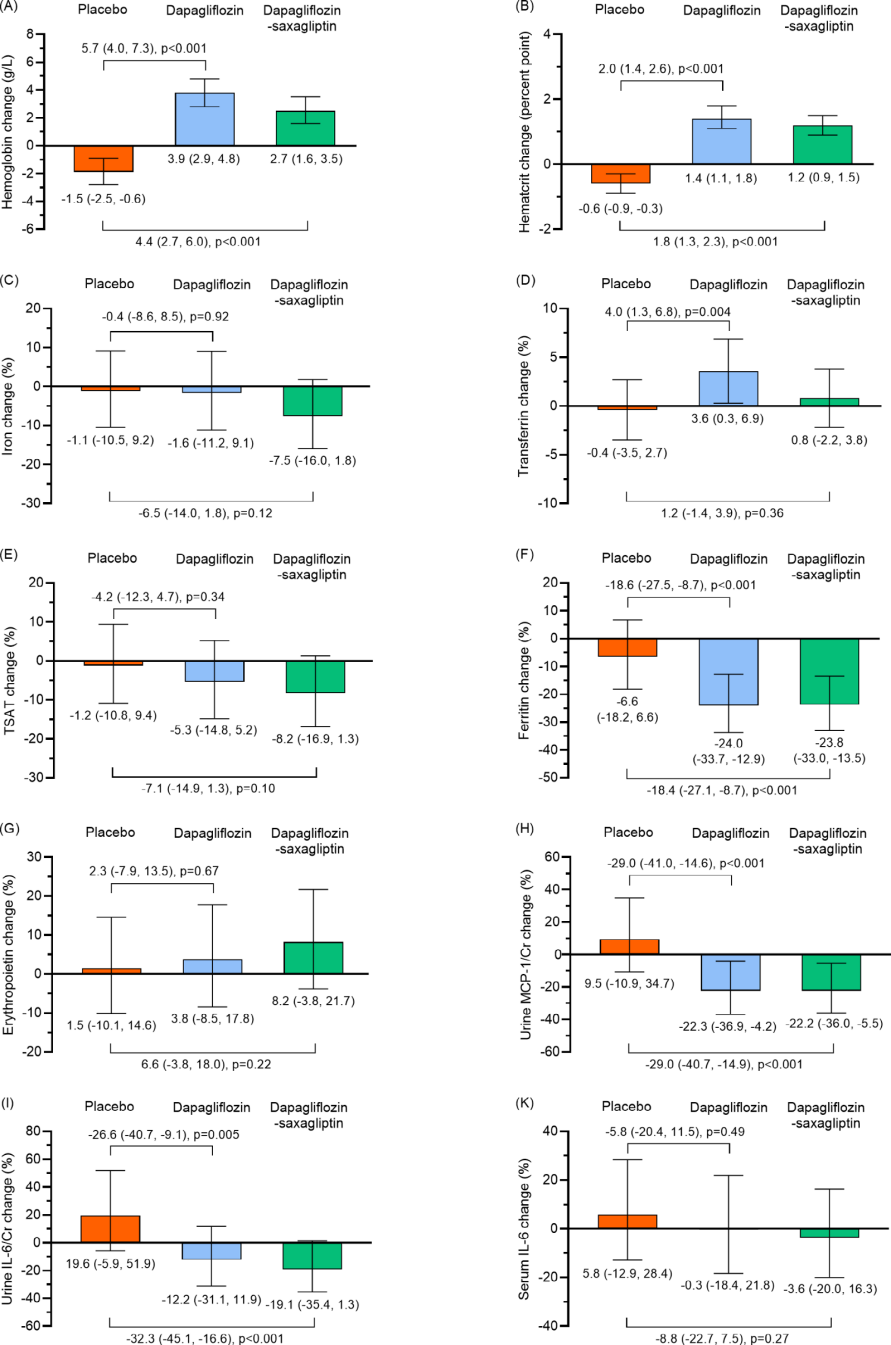
Changes in markers of hematopoiesis, iron metabolism and inflammation. Mean absolute changes in (**A**) hemoglobin and (**B**) hematocrit over time. Mean percentage changes in (**C**) serum iron, (**D**) transferrin, (**E**) TSAT, (**F**) ferritin, (**G**) erythropoietin, (**H**) urinary MCP-1/Cr, (**J**) urinary IL-6/Cr and (**K**) serum IL-6. Numbers across bars represent between group differences in change relative to placebo group. Cr, creatinine; MCP-1, monocyte chemoattractant protein; IL-6, interleukin-6; TSAT, transferrin saturation

Changes in biomarkers by treatment allocation and their differences relative to placebo are presented in Fig. 1. In the placebo group, hemoglobin decreased during the study period (mean change − 1.5 g/L [-2.5, -0.6], p < 0.001). Compared to placebo, dapagliflozin and dapagliflozin-saxagliptin increased hemoglobin by 5.7 g/L (4.0, 7.3), p < 0.001, and 4.4 g/L (2.7, 6.0), p < 0.001, respectively. There was no clear difference in hemoglobin change between dapagliflozin and dapagliflozin-saxagliptin (-1.3 g/L [-2.9, 0.34], p = 0.16) Similarly, both treatments also increased hematocrit compared to placebo.

There was a significant reduction in ferritin in both dapagliflozin alone and dapagliflozin-saxagliptin group compared to placebo (-18.6% [-27.5, -8.7], p < 0.001 and − 18.4% [-27.1, -8.7], p < 0.001, respectively) with no difference in iron concentration (Fig. 1). Transferrin increased in the dapagliflozin group (4.0% [1.3, 6.8], p = 0.004) compared to placebo, but not in the dapagliflozin-saxagliptin group (1.2% [-1.4, 3.9], p = 0.36). Accordingly, the increase in transferrin in dapagliflozin was higher than that in dapagliflozin-saxagliptin (2.7% [0.1,5.2], p = 0.04) There were numerical trends to decrease TSAT and increase EPO among patients treated with dapagliflozin, although these were not statistically significant. There was no clear difference between dapagliflozin and dapagliflozin saxagliptin on iron, TSAT, ferritin and EPO (all p values > = 0.15).

Dapagliflozin reduced urinary inflammatory markers (Fig. 1). Between-group differences in urinary MCP-1/Cr change versus placebo were − 29.0% (-41.0, -14.6), p < 0.001, and − 29.0% (-40.7, -14.9), p < 0.001 for dapagliflozin alone and the dapagliflozin-saxagliptin group, respectively. Changes in urinary IL-6/Cr with dapagliflozin and dapagliflozin-saxagliptin were − 26.6% (-40.7, -9.1), p = 0.005, and − 32.3% (-45.1, -16.6), p < 0.001, respectively. Serum IL-6 did not change in each treatment group. There was no difference in the effect on MCP-1/Cr, IL-6/Cr and serum IL-6 between dapagliflozin and dapagliflozin-saxagliptin (all p values > = 0.44).

Correlation coefficients between changes in makers from baseline to week 24 by treatment were shown in Table 2. Negative correlations between changes in serum IL-6 and iron and TSAT were observed in all treatment groups (all p ≤ 0.01). In the dapagliflozin group, decreases in urinary MCP-1/Cr and IL-6/Cr were correlated with increases in iron and transferrin. Urinary IL-6/Cr change was correlated with changes in TSAT and erythropoietin only in the dapagliflozin group.

**Table 2:** Correlation coefficients between changes in inflammation markers and those in iron markers and erythropoietin from baseline to week 24 by treatment group

Biomarkers changes	Placebo		Dapagliflozin		Dapagliflozin-saxagliptin	
	Correlation coefficient	p value	Correlation coefficient	p value	Correlation coefficient	p value
**urinary MCP-1/Cr change**						
Iron change	0.04	0.72	-0.26	0.01	0.00	0.97
Transferrin change	0.04	0.68	-0.35	< 0.001	0.05	0.59
TSAT change	0.03	0.81	-0.17	0.12	-0.02	0.84
Ferritin change	0.17	0.12	0.15	0.15	-0.10	0.30
Erythropoietin change	-0.06	0.61	0.13	0.22	0.05	0.62
**urinary IL-6/Cr change**						
Iron change	0.11	0.32	-0.37	< 0.001	0.04	0.69
Transferrin change	0.13	0.22	-0.24	0.02	0.11	0.30
TSAT change	0.07	0.52	-0.30	0.004	0.01	0.94
Ferritin change	0.02	0.83	0.02	0.84	0.04	0.68
Erythropoietin change	0.07	0.54	0.27	0.01	0.01	0.89
**serum IL-6 change**						
Iron change	-0.33	< 0.001	-0.37	< 0.001	-0.29	0.002
Transferrin change	0.05	0.63	-0.20	0.04	-0.14	0.12
TSAT change	-0.34	< 0.001	-0.32	< 0.001	-0.23	0.01
Ferritin change	0.04	0.66	0.18	0.06	0.03	0.73
Erythropoietin change	-0.01	0.92	0.05	0.63	0.26	0.004

Correlation coefficients were shown as Pearson`s R

Cr, creatinine; MCP-1, monocyte chemoattractant protein; IL-6, interleukin-6; TSAT, transferrin saturation

## Discussion

In this post-hoc analysis of the DELIGHT trial, dapagliflozin and a combination of dapagliflozin and saxagliptin increased hemoglobin/hematocrit and reduced ferritin in patients with type 2 diabetes and CKD. Dapagliflozin reduced urinary inflammatory markers, specifically MCP-1/Cr and IL-6/Cr.

The effect on iron homeostasis with SGLT2 inhibition has been assessed in patients with heart failure [7, 8]. We confirm that dapagliflozin reduces ferritin in patients with CKD and type 2 diabetes in whom anemia and disordered iron metabolism is highly prevalent [1]. Impacts on other markers, including numerical decrease in TSAT and increase in EPO, were also similar to those in other studies [7, 8]. Together with increases in hemoglobin and hematocrit, which cannot be explained only by hemoconcentration [15, 16], these results suggest that SGLT2 inhibitors might increase iron mobilization from intracellular storage. Iron utilization with SGLT2 inhibition is hypothesized to contribute to its cardiovascular protection via increased cytosolic iron and enhanced ATP production in the myocardium independent of increases in hemoglobin/hematocrit [17].

Dapagliflozin with and without saxagliptin reduced urinary inflammation markers of MCP-1/Cr and IL-6/Cr. Inflammation is strongly associated with diabetic kidney disease progression as well as cardiovascular events [18]. In preclinical models of diabetes, SGLT2 inhibition reduced hyperglycemia-induced oxidative stress and advanced glycation end products within proximal tubular cells and attenuated tubulointerstitial inflammation and fibrosis [5]. Although further study is warranted to assess how much the anti-inflammatory properties of SGLT2 inhibitor contributes kidney protection, an exploratory mediation analysis of a cardiovascular outcome trial reported that the effect of canagliflozin on urinary MCP-1 partly mediated reduction in kidney injury morecule-1, a marker of kidney damage, in patients with type 2 diabetes and CKD [19].

The current study does not support the additive effect of saxagliptin on markers of hematopoiesis, iron and inflammation when used in combination with SGLT2 inhibitors. In the current study, the reduction in HbA1c was more pronounced in the combination dapagiliflozin-saxigliptin group compared to dapagliflozin alone, suggesting that the decreases in iron and inflammation markers with dapagliflozin observed in this study are unlikely mediated by improved glycemic control.

Increased systemic inflammation worsens iron availability, partly via increased hepcidin [1]. The observed negative correlations between serum IL-6 and iron and TSAT at baseline, and the association between changes in serum/urine IL-6 and TSAT during dapagliflozin treatment were consistent with this notion. Although the effect of dapagliflozin on serum IL-6 was not significant in DELIGHT, the effect size was similar to data from a larger clinical trial with canagliflozin demonstrating a 5% decrease in plasma IL-6 in patients with type 2 diabetes at high cardiovascular risk [12]. Reductions in urinary inflammation markers were also correlated with increases in iron markers only in the dapagliflozin group. These data suggest an important link between SGLT2 inhibition, inflammation, erythropoiesis, and iron utilization, although they do not allow for conclusions about causality.

Limitations of this study include its post-hoc nature, which may increase chance findings. Secondly, the absence of hepcidin concentration precludes our ability to test the hypothesis that SGLT2 inhibition addresses functional iron deficiency by reducing hepcidin. Third, these analyses do not account for use of iron preparation or ESA therapy during the study. Possible unbalance in anemia treatment could bias the effect estimation on hematological and iron parameters, although the short follow up duration of 6 moths reduced that risk. Finally, urinary MCP-1 and IL-6 were measured in spot urine samples and indexed to urinary creatinine. This might decrease the precision of the observed effect estimates as the concentration of urinary analytes are more variable in spot urine than 24-hour samples. However, despite the potential decrease in statistical power, we observed statistically significant reduction in urinary inflammation markers.

## Conclusions

In patients with type 2 diabetes and CKD, dapagliflozin increased hemoglobin/hematocrit and reduced ferritin and urinary MCP-1/Cr and IL-6/Cr, suggesting potentially important effects on iron metabolism and inflammation.

### Electronic supplementary material

Below is the link to the electronic supplementary material.

Supplementary Material 1

## Data Availability

Individual de-identified participant data are not freely available because of the risk of patient re-identification, but interested parties can request access to de-identified participant data or anonymised clinical study reports through submission of a request for access via the AstraZeneca Group of Companies Data Request Portal.

## Abbreviations

CKD: Chronic kidney disease
EPO: Erythropoietin
DPP-4: Dipeptidyl peptidase-4
IL-6: Interleukin-6
MCP-1: Monocyte chemoattractant protein-1
SGLT2: Sodium glucose co-transporter 2
TSAT: Transferrin saturation
UACR: Urinary albumin-creatinine ratio
