# Supplementary material for: Effects of dapagliflozin and dapagliflozin-saxagliptin on erythropoiesis, iron and inflammation markers in patients with type 2 diabetes and chronic kidney disease: data from the DELIGHT trial
Source: Cardiovasc Diabetol. 2023 Nov 28;22:330. doi: 10.1186/s12933-023-02027-8 (PMC10685512; doi:10.1186/s12933-023-02027-8)
Supplement: Supplementary file 1 — Supplementary Material 1 [file 12933_2023_2027_MOESM1_ESM.pdf]

Additional file 1: Supplementary table S1. Correlation coefficients between inflammation and iron markers and erythropoietin at baseline in overall population

	Urinary MCP-1/Cr Correlation coefficient	p value	Urinary IL-6/Cr Correlation coefficient	p value	Serum IL-6 Correlation coefficient	p value
Iron	-0.12	0.05	-0.02	0.73	-0.18	<0.01
Transferrin	-0.16	0.01	-0.10	0.11	-0.07	0.17
TSAT	-0.04	0.51	0.04	0.54	-0.14	0.008
Ferritin	0.03	0.61	0.06	0.30	-0.07	0.20
Erythropoietin	0.05	0.42	-0.02	0.76	0.05	0.39

Correlation coefficients were shown as Pearson`s R.

Cr, creatinine; MCP-1, monocyte chemoattractant protein; IL-6, interleukin-6; TSAT, transferrin saturation
